# Ceramic and Composite Polishing Systems for Milled Lithium Disilicate Restorative Materials: A 2D and 3D Comparative In Vitro Study

**DOI:** 10.3390/ma15155402

**Published:** 2022-08-05

**Authors:** Carlos A. Jurado, Clarisa Amarillas-Gastelum, Kelvin I. Afrashtehfar, Liliana Argueta-Figueroa, Nicholas G. Fischer, Abdulrahman Alshabib

**Affiliations:** 1 Woody L. Hunt School of Dental Medicine, Texas Tech University Health Sciences Center, El Paso, TX 79905, USA; 2 Department of General Dentistry, Stony Brook University School of Dentistry, Stony Brook, NY 11794, USA; 3 Clinical Sciences Department, College of Dentistry, Ajman University, Ajman City P.O. Box 346, United Arab Emirates; 4 Department of Reconstructive Dentistry and Gerodontology, School of Dental Medicine, University of Bern, 3010 Berne, Switzerland; 5 School of Dentistry, Universidad Benito Juarez de Oaxaca, Oaxaca 68120, Mexico; 6 Minnesota Dental Research Center for Biomaterials and Biomechanics, University of Minnesota School of Dentistry, Minneapolis, MN 55455, USA; 7 Department of Restorative Dentistry, King Saud University College of Dentistry, Riyadh 12372, Saudi Arabia; 8 Engineer Abdullah Bugshan Research Chair for Dental and Oral Rehabilitation, King Saud University, Riyadh 11545, Saudi Arabia

**Keywords:** CAD-CAM, disilicate lithium, dental polishing, dental prosthesis, esthetic dentistry

## Abstract

***Purpose:*** This study aims to evaluate the effectiveness of two ceramic and two composite polishing systems for a novel chairside computer-aided design/computer-aided manufacturing (CAD/CAM) lithium disilicate ceramic with three-dimensional and two-dimensional microscopy images. This ceramic material can be used for implant-supported or tooth-borne single-unit prostheses. ***Materials and Methods:*** Sixty flat samples of novel chairside CAD/CAM reinforced lithium disilicate ceramic (Amber Mill, Hass Bio) were divided into five groups (n = 15/group) and treated as follows: Group 1 (NoP), no polished treatment; group 2 (CeDi), polished with ceramic Dialite LD (Brasseler USA); group 3, (CeOp) polished with ceramic OptraFine (Ivoclar Vivadent); group 4, (CoDi) polished with composite DiaComp (Brasseler USA), and group 5 (CoAs), polished with composite Astropol (Ivoclar Vivadent). The polished ceramic surface topography was observed and measured with three-dimensional and two-dimensional images. ***Results:*** All polishing systems significantly reduced the surface roughness compared with the non-polished control group (Sa 1.15 μm). Group 2 (CeDi) provided the smoothest surface arithmetical mean eight with 0.32 μm, followed by group 3 (CeOp) with 0.34 μm. Group 5 (CoAs) with 0.52 μm provided the smoothest surface among the composite polishing kits. Group 4 (CoDi) with 0.66 μm provided the least smooth surface among all polishing systems tested. ***Conclusions:*** Despite the effectiveness of ceramic polishing systems being superior to composite polishing systems of the CAD/CAM lithium disilicate restorative material, both polishing systems significantly improved the smoothness.

## 1. Introduction

The development of computer-aided design and computer-aided manufacturing (CAD/CAM) dental systems started in the 1970s and the first dental CAD/CAM restoration was manufactured in 1983 [1,2]. Dr. Duret fabricated the first inlay restoration, which was milled out of a feldspathic ceramic, and the first dental posterior crown in 1985 [3]. Dental CAD/CAM systems have greatly evolved from monitors with two-dimensional to three-dimensional images and from simple inlay restorations to restorations such as implants and full mouth reconstructions [4,5,6,7]. Dental CAD/CAM technology offers a wide variety of composites, ceramics, and combinations [8,9].

Dental treatments with CAD/CAM technology can fulfill patient esthetic demands from single to multiple ceramic restorations [10,11]. Lithium disilicate is a well-known dental ceramic with high esthetics and fracture resistance [12,13]. Lithium disilicate (SiO_2_-Li_2_O) was first introduced as a core material in the late 1900s and was originally fabricated by heat-pressing ingots, similar to a lost-wax technique for traditional alloys [14,15]. This material was replaced by updated materials, such as IPS e.max (Ivoclar Vivadent) in 2009 with smaller and more uniform crystals, which reduced complications such as chipping and fractures [16,17]. Similarly, the CAD/CAM version of lithium disilicate (e.max CAD, Ivoclar Vivadent, Schaan, Liechtenstein) was introduced in 2006 and was fabricated in a “blue stage” or pre-sintered stage requiring firing processing after milling before cementation [18,19]. A range of materials exists today with different shades and grades of translucency depending on the size and density of the crystals [20].

Companies have developed newer reinforced lithium disilicate claiming to improve mechanical and esthetic properties with the increased acceptance and demand for CAD/CAM lithium disilicate. Recently, a novel pre-sintered CAD/CAM lithium disilicate (Amber Mill from HASS Bio, Gangneung, Gangwon-do, Korea) was introduced to the market and, according to the manufacturer, it has increased mechanical and esthetic properties in comparison to early lithium disilicate materials [21]. Traditional lithium disilicate (e.max CAD, Ivoclar Vivadent) has a composition of 59% LiSi_2_O_5_, 33% glass, and crystal sizes of 2 to 4 µm, and the novel lithium disilicate (Amber Mill, Hass Bio) presents 46.1% LiSi_2_O_5_, 33.7% and crystal sizes size of 0.2 µm [22]. This novel material controls the level of translucency, such as high, medium, or low, within the single block based on the temperature selected during the sintering process. It has been recommended to have smooth polished ceramic restorations for many reasons [22,23,24]. Polished surfaces provide little retention for bacteria and thus reduce the exposure to plaque formation and prevent adverse effects on periodontal tissues [25,26,27,28]. Moreover, smooth surfaces are less likely to stain and avoid negatively impacting the esthetic results [29,30]. When manufacturing CAD/CAM ceramic crowns, it is recommended to follow manufacturers’ recommendations to maximize favorable results, such as using specific polishing systems. Unfortunately, not all companies manufacturing dental ceramics recommend or manufacture polishing systems, and clinicians need to find polishing systems from different brands [31,32]. A novel chairside CAD/CAM lithium disilicate (Amber Mill; HASS Bio, Fairfax, VA, USA) does not include its own polishing system. Therefore, the aim of the study is to compare two ceramic and two polishing systems for this novel lithium disilicate ceramic. The first null hypothesis was that ceramic and composite polishing systems provide smoother surfaces than non-polished samples. The second null hypothesis was that there is no difference in smoother surfaces among ceramic and composite polisher systems.

## 2. Materials and Methods

The sample size was determined based on a previous study [33]. Thus, 75 flat samples (10.44 mm width, 12.5 mm length, 15 mm height, and 2 mm thick) were obtained from the chairside CAD/CAM lithium disilicate (HASS Bio, Gangwon-do, Korea) ceramic blocks for surface evaluation. Samples were sintered following the manufacturer’s recommendation at 400 °C for 30 min, and they were assigned to 5 groups (n = 15 ^ea^/group) as follows: group 1 (NoP), no polished treatment; group 2 (CeDi), polished with ceramic polishing kit Dialite LD (Brasseler USA, Savannah, GA, USA); group 3 (CeOp), polished with ceramic polishing kit OptraFine (Ivoclar Vivadent, Schaan Liechtenstein); group 4 (CoDi), polished with composite polishing kit DiaComp (Brasseler USA, Savannah, GA, USA), and group 5 (CoAs), polished with composite polishing kit Astropol (Ivoclar Vivadent, Schaan, Liechtenstein). Each polishing treatment was applied for 2 min following the manufacturer’s instructions by a single operator. The materials and methods are summarized in Table 1.

Samples received an ultrasonic bath (UltraMet 2002, Buehler Corp, IL, USA) cleaning with isopropyl alcohol 98% solution for 10 min, followed by surface evaluation aided by a 3D laser confocal scanning microscope (Keyence VHX-6000, Keyence, Itasca, IL, USA). Samples were scanned and measured by a vertical scan using a Å~5 magnification lens with a 1 mm × 1 mm field of view. The surface arithmetical mean height (Sa), maximum surface height (Sz), surface texture aspect ratio (Str), surface arithmetical mean peak curvature (Spc), and the surface developed interfacial area ratio (Sdr). Sa is a common measure of surface roughness, and Sz is the sum of surface’s largest peak height and pit depth. Str is a measure of surface uniformity, where values close to 1.0 are highly uniform versus values close to 0.0 are more random.

Conversely, smaller Spc values indicate rounder shapes on a surface vs. larger values indicate more pointy shapes. Finally, Sdr expresses the percentage of an additional surface area contributed by the surface texturing and topography compared to a planar area. Two- (2D) and three-dimensional (3D) images were also obtained with the laser scanner for qualitative comparison.

Data are presented as median and interquartile ranges. Kolmogorov–Smirnov test determined the non-parametrically distributed data. Kruskal–Wallis and Mann–Whitney U tests, with a Bonferroni correction, were used to compare groups. All statistical analyses were performed using statistical software (SPSS ver. 25, IBM Corp., Armonk, NY, USA). The level of significance was set to α = 0.008.

## 3. Results

The comparison of ceramic and composite polishing kits for chairside CAD/CAM lithium disilicate is shown in Table 2. Group 1 (NoP), the control group with no polishing treatment, displayed the highest surface roughness (Sa; 1.15 μm), followed by both groups polished with resin composite polishing kits [group 4 (CoDi) at 0.66 μm and group 5 (CoAs) with 0.52 μm]. Ceramic polishing kits displayed the lowest Sa values [2 (CeDi) at 0.32 μm and 3 (CeOp) 0.34 μm]. Similar trends were seen with surface maximum height (Sz) where group NoP showed significantly higher values than all other groups and the composite polishing groups [4 (CoDi) and 5 (CoAs) produced higher values than ceramic polishing methods.

**Figure 1:**
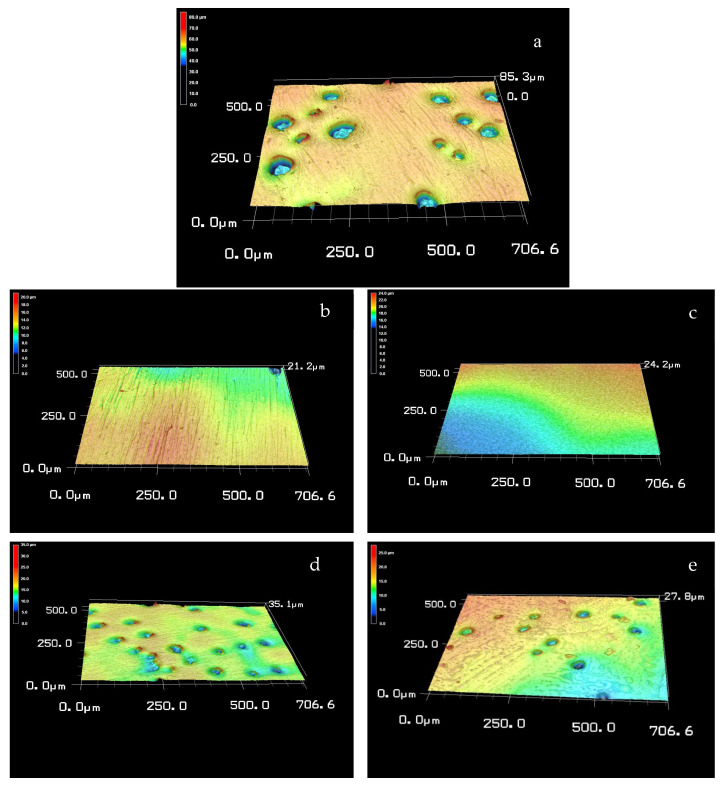
Three-dimensional images of chairside CAD/CAM reinforced lithium disilicate ceramic; (**a**) non-polished; (**b**) polished with Dialite ceramic polishing; (**c**) polished with OptraFine ceramic polishing; (**d**) polished with Diacomp composite polishing; (**e**) polished with Astropol composite polishing.

**Figure 2:**
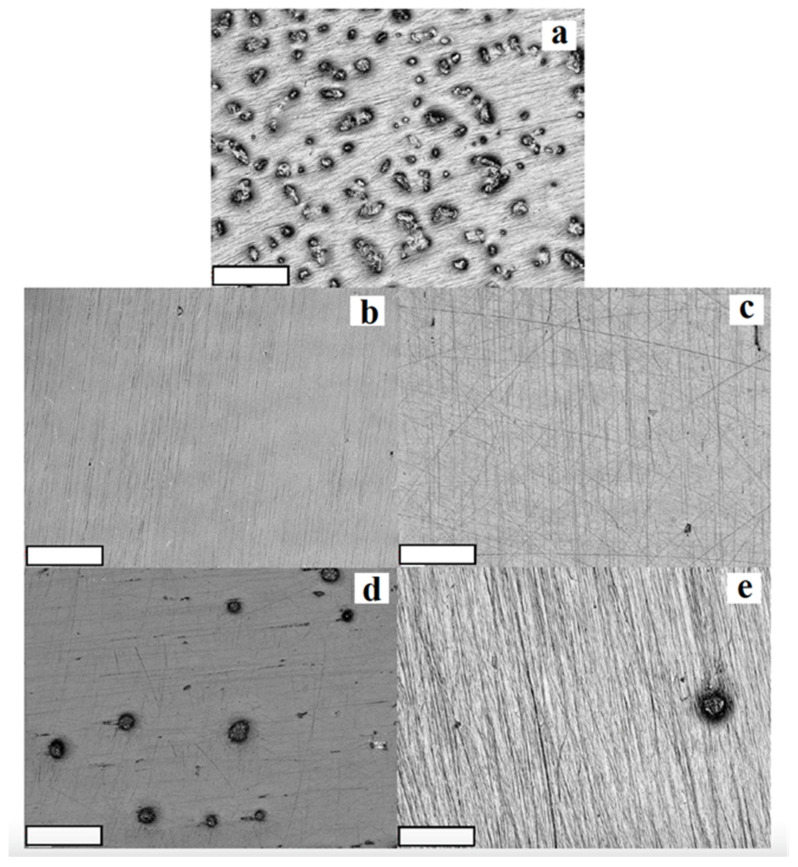
Scanning electron microscopy images of chairside CAD/CAM reinforced lithium disilicate ceramic; (**a**) non-polished; (**b**) polished with Dialite ceramic polishing; (**c**) polished with OptraFine ceramic polishing; (**d**) polished with Diacomp composite polishing; (**e**) polished with Astropol composite polishing.

Surface texture aspect ratio (Str) results showed the highest values for group 1 (NoP) with statistically significant reduction seen after polishing with group 2 (CeDi) and group 5 (CoAs). Surface arithmetic mean peak curvature (Spc) results showed that only group 2 (CeDi) and group 3 (CeOp) changed values from group 1 (NoP) control: group 2 (CeDi) significantly increased whereas group 3 (CeOp) significantly decreased. All groups showed similar surface-developed interfacial area ratios (Sdr). Two-dimensional and three-dimensional images are shown in Figure 1 and Figure 2. The images clearly display rougher group 1 (NoP) surfaces than the rest of the groups. Surfaces polished with ceramic polishing kits (groups 2 and 3) tended to produce visually smoother surfaces than surfaces polished with composite polishing kits (groups 4 and 5).

## 4. Discussion

The 3D laser confocal scanning microscope utilized in this study provides three-dimensional topographic observations to evaluate different surface aspects, such as the spatial arithmetical mean height (Sa), which is the extension of the arithmetical mean height of a line to a surface. It expresses, as an absolute value, the difference in height of each point compared to the arithmetical mean of the surface. This parameter is commonly used to evaluate surface roughness where rougher surfaces produce higher Sa values [34,35]. We also evaluated the maximum height (Sz), the sum of the largest peak height value, and the largest pit depth value within the defined area. The uniformity of the surface texture ratio (Str) is a measure of the spatial isotropy or directionality of the surface texture and was another aspect evaluated. The arithmetic means peak curvature (Spc), and the arithmetic mean of the principal curvature of the peaks on the surface, were also assessed. Lastly, the developed interfacial area ratio (Sdr) is expressed as the percentage of the definition area’s additional surface contributed by the texture compared to the planar definition area [36]. Moreover, the surface of the ceramic was also evaluated with 2D images that provide qualitative analysis of the morphology unable to be seen with the naked eye [33]. In the present study, the surface roughness with a 3D microscope that provides all the mentioned parameters plus 2D imaging was also taken to evaluate more information regarding the topography of the materials tested. Three-dimensional imaging is advantageous compared to traditional two-dimensional because it provides quantitative information compared to only qualitative 2D images [34]. However, they can complement and correlate with one another, but both are good visual metrics of the morphology of the surface [35].

Amber Mills (Hass Bio) is a new lithium disilicate available in the market. Despite it falling into the lithium disilicate ceramic category, it is microstructurally different from popular products, (e.g., e.max CAD). This novel lithium disilicate contains less Li_2_Si_2_O_2_ percentage (46%) than the traditional (62%) but higher glass material (33% for Amber Mill; 29% for e.max CAD) [36]. Amber Mills contains 13% quartz; however, traditional lithium disilicate does not contain it, and the microstructural appearance and characteristics of the novel lithium disilicate are similar to zirconia-reinforced lithium disilicate ceramics (Suprinity PC and Celtra Duo) due to the submicrometric Li_s_Si_2_O_5_ phase [22,37]. This different microstructural composition of the novel material provides unexplored outcomes in surface roughness. In our study, we evaluated the result of different polishing systems in this novel lithium disilicate with a modified microstructural appearance, and the smoothness achieved by two ceramic polishing kits and two composite polishing kits was investigated. The results obtained in this study displayed that group 2 (CeDi) with ceramic polishing provided the lowest roughness mean height of 0.32 μm, followed by group 3 (CeOp) at 0.34 μm which is also a ceramic polishing kit. Both composite polishing kits, group 4 (CoDi) at 0.66 μm and 5 (CoAs) at 0.53 μm, provided a smoother surface than control group 1 (NoP) at 1.15 μm, but higher roughness than ceramic groups. Thus, the first null hypothesis of the study “ceramic and composite polishing systems provide smoother than non-polished samples” was accepted. A previous study has demonstrated that regular enamel roughness surface ranges from 0.45 to 0.65 μm [38]. The ability of the ceramic polishing kits to achieve lower values than enamel is likely beneficial to reducing plaque formation and preventing detrimental effects on periodontal tissues.

Both ceramic polishing systems group 2 (CeDi) and group 3 (CeOp) provided smoother surfaces than both composite polishing systems group 4 (CoDi) and group 5 (CoAs). These differences were statistically significant, so it can thus be concluded that the ceramic and composite produce dissimilar results. Therefore, the second null hypothesis of the study “there is no difference in smoother surface among ceramic and composite polisher systems” was rejected. Even though the surface polished by resin composites kits displayed higher roughness than the ceramic counterparts, the surface of the composite was smoother than the control group with no polishing provided. A previous study evaluating composite polishers on different resins concluded that surface smoothness is material-dependent rather than step-dependent, but all composite systems are clinically acceptable [39]. Unfortunately, minimal data comparing composite and ceramic polishing systems for CAD/CAM ceramics are available. Even though the studies comparing composite and ceramic polishers for ceramics are scarce, our findings concur with some other studies comparing polishers on resin ceramics and resin infiltrated ceramics. First, a previous study compared composite and ceramic polishing systems on feldspathic ceramic and resin ceramics and concluded that ceramic and composite kits could satisfactorily be used for resin ceramic restorations [39]. Furthermore, another study compared ceramic and composite polishing systems for resin infiltrated ceramic and concluded that all polishers improved the surface in comparison with “non-polished” samples (control group), but composite polishers were less reliable than ceramic polishing [23].

The results of this study provided surface roughness values in the range of 0.32–0.66 µm and Kaplan et al. reported that values < 10 µm are clinically undetectable; therefore, the polished specimens achieved acceptable surface finish. Comparing ceramic and composite polishing systems on ceramics or composite has been performed before but not specifically for chair-side CAD/CAM lithium disilicate like in our study [23,40,41]. One of the concepts behind this comparison is clinically oriented because if dental clinicians do not have access to the recommended ceramic polishing system, the question rising is how effective can a composite system be compared with the ideal or even with no polishing treatment provided. Moreover, this study can also be educational for dental students questioning how critical it is to use the indicated tools for the surface polishing of the restoration.

In this study, two composite and two ceramic polishing systems were compared but there are many more brands available in the market. Thus, further studies are required to compare more novel brands and traditional ones to verify their efficiency. Moreover, we did not provide glazing treatment in our study, as may be performed by some clinicians. However, it is also important to mention that a recent study comparing polishing techniques and glazing treatment on CAD/CAM ceramics concluded that polishing techniques provide better results than the glazing methods for surface roughness regardless of the dental ceramic type [40,42]. Nevertheless, it could be interesting to have in vitro and in vivo investigations comparing polishing and glazing for this novel dental ceramic.

## 5. Conclusions

Ceramic and composite polishing systems significantly improve the surface smoothness of a novel chairside CAD/CAM lithium disilicate ceramic (Amber Mill, Hass Bio). However, ceramic polishing systems are recommended as their polishing effectiveness was higher than the composite polishing systems.

## Figures and Tables

**Table 1:** Description of polishing systems used in this study.

Group	Polishing System	Manufacturer	Components	Manufacturer Instructions
Group 1 (NoP)	No polishing	None	None	None
Group 2 Ceramic System (CeDi)	Dialite LD for lithium disilicate (Brasseler USA)	Brasseler USA, Savannah, GA, USA.	Medium (red) and fine (yellow) grits (red) for: - occlusal surface (points). - labial surfaces (cones). - interproximal (fine knife edge).	Medium grifts (red) for pre-polishing and fine grifts (yellow) for final polishing. Speed: 5000–7000 rpms. May be used dry with a feather touch or wet.
Group 3 Ceramic System (CeOp)	OptraFine (Ivoclar Vivadent)	Ivoclar Vivadent, Schaan, Liechtenstein.	- Light blue diamond finishers (F) in flame, cup, and disc shapes. - Dark blue diamond polishers (P) in flame, cup, and disc shapes. - Nylon brushes for high-gloss polishing (HP), suitable for use in conjunction with the paste. - Diamond polishing paste for high-gloss polishing (HP).	Step 1: Prep polishing using “light blue”. Step 2: Polishing using “dark blue”. Step 3: High-gloss polishing with brushes and polishing paste. Speed: 1000 up to max 1500 rpm for diamond finishers (F) and polishers (P) with copious water spray (>50 mL/min). 7000 up to max 1000 rpm for brushes (HP) and polishing paste without water spray.
Group 4 Composite System (CoDi)	DiaComp (Brasseler USA)	Brasseler USA, Savannah, GA, USA.	Medium (green) and fine (gray) grits for: - Points for occlusal surfaces. - Knife-edge for proximal, buccal, and lingual. - Cup for following the contour of the tooth and restoration.	Green medium grit is used to remove scratches and satin shine surfaces. Gray fine grit leaves a high shine finish. Speed: 5000 to 6000 rpm. May be used dry with a feather touch or wet.
Group 5 Composite System (CoAs)	Astropol (Ivoclar Vivadent)	Ivoclar Vivadent, Schaan, Liechtenstein.	Astropol F (grey), P (green) and HP (pink).Shapes: - Small flame, large flame, cup, and disc.	Astropol F (grey) for removal of excess composite material. Astropol P (green) for polishing. Astropol HP (pink) for final high-gloss. Speed: 7000 to 10,000 rpm. Only used with copious water spray (>50 mL/min). Use without polishing paste.

Abbreviations: NoP, no polishing; CeDi, ceramic Dialite; CeOp, ceramic OptraFine; CoDi, composite DiaComp; CoAs, composite Astropol.

**Table 2:** Results for comparing ceramic and composite polishing systems for chairside CAD/CAM reinforced lithium disilicate ceramic.

Factors	Groups				
	Group 1 No Polishing (μm)	Ceramic		Composite	
		Group 2 Dialite LD (μm)	Group 3 OptraFine (μm)	Group 4 DiaComp (μm)	Group 5 Astropol (μm)
**Sa** **(Surface arithmetical mean height)**	1.15 (0.81, 1.29)	0.32 (0.20, 0.50)	0.34 (0.25, 0.40)	0.66 (0.39, 0.85)	0.52 (0.45, 0.67)
**Sz** **(Surface maximum height)**	11.41 (5.23, 11.89)	3.59 (3.27, 4.57)	2.81 (2.39, 3.44)	6.43 (2.70, 10.90)	4.08 (3.19, 7.64)
**Str** **(Surface texture aspect ratio)**	0.57 (0.44, 0.73)	0.37 (0.20, 0.47)	0.46 (0.35, 0.53)	0.52 (0.40, 0.66)	0.39 (0.31, 0.54)
**Spc** **(Surface arithmetic mean peak curvature)**	2861 (2536, 3132)	3314 (2926, 3528)	2342 (2128, 2512)	2851 (2622, 3204)	2845 (2705, 3085)
**Sdr** **(Surface developed interfacial area ratio)**	0.04 (0.03, 0.05)	0.05 (0.04, 0.07)	0.02 (0.02, 0.03)	0.03 (0.03, 0.06)	0.03 (0.03, 0.03)

Data are shown as median (interquartile range). Dissimilar letter indicates statistical significance difference between groups for each factor, as described in the Materials and Methods.

## Data Availability

Not applicable.
